# Ether Lipids in Obesity: From Cells to Population Studies

**DOI:** 10.3389/fphys.2022.841278

**Published:** 2022-03-03

**Authors:** Yvette L. Schooneveldt, Sudip Paul, Anna C. Calkin, Peter J. Meikle

**Affiliations:** ^1^Baker Heart and Diabetes Institute, Melbourne, VIC, Australia; ^2^Central Clinical School, Faculty of Medicine, Nursing & Health Sciences, Monash University, Melbourne, VIC, Australia; ^3^Baker Department of Cardiometabolic Health, University of Melbourne, Parkville, VIC, Australia

**Keywords:** plasmalogens, adipose tissue, obesity, alkylglycerols, ether lipids

## Abstract

Ether lipids are a unique class of glycero- and glycerophospho-lipid that carry an ether or vinyl ether linked fatty alcohol at the *sn*-1 position of the glycerol backbone. These specialised lipids are important endogenous anti-oxidants with additional roles in regulating membrane fluidity and dynamics, intracellular signalling, immunomodulation and cholesterol metabolism. Lipidomic profiling of human population cohorts has identified new associations between reduced circulatory plasmalogen levels, an abundant and biologically active sub-class of ether lipids, with obesity and body-mass index. These findings align with the growing body of work exploring novel roles for ether lipids within adipose tissue. In this regard, ether lipids have now been linked to facilitating lipid droplet formation, regulating thermogenesis and mediating beiging of white adipose tissue in early life. This review will assess recent findings in both population studies and studies using cell and animal models to delineate the functional and protective roles of ether lipids in the setting of obesity. We will also discuss the therapeutic potential of ether lipid supplementation to attenuate diet-induced obesity.

## Ether Lipids

### Structure and Biological Functions

**FIGURE 1:**
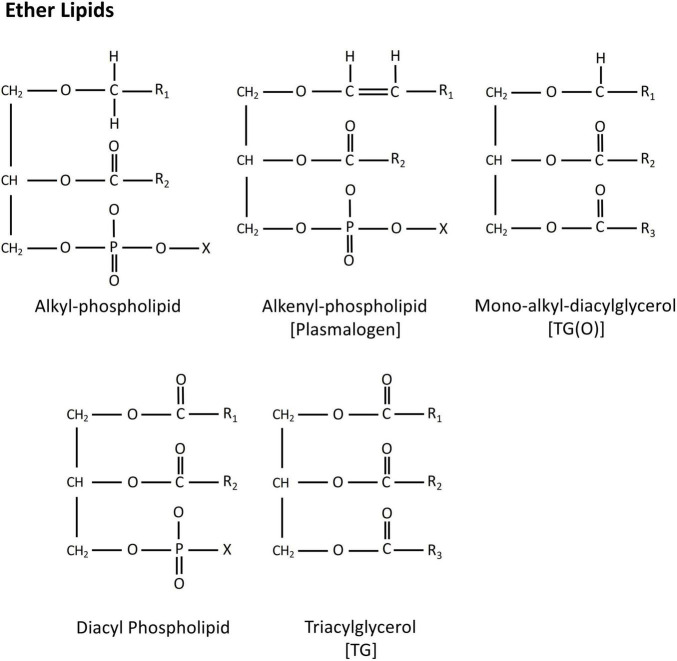
Chemical structure of alkyl-, alkenyl- and mono-alkyl-ether lipids. Diacyl phospholipid and triacylglycerol exhibits typical structure of a glycerol-lipids.

Ether lipids are a unique class of peroxisome-derived glycero- and glycerophospho-lipid. They carry an ether or vinyl ether linked fatty alcohol at the *sn*-1 position, and an ester linked fatty acid either at the *sn*-2 position (ether phospholipids), or at both the *sn*-2 and *sn*-3 positions (ether glycerolipids). This is contrary to conventional glycerol-based lipids that have acyl chains attached by an ester linkage at the *sn*-1 position (Figure 1). To date, ether analogs of triacyclglycerols [mono-alkyl-diacylglycerols, TG(O)] and various phospholipid classes, including phosphatidylethanolamine [alkyl-phosphatidylethanolamine, PE(O)], phosphatidylcholine [alkyl-phosphatidylcholine, PC(O)], phosphatidylinositol [alkyl-phosphatidylinositol, PI(O)] and phosphatidylserine [alkyl-phosphatidylserine, PS(O)] have been reported (Nagan and Zoeller, 2001; Ivanova et al., 2012; Nagy et al., 2012; Ma et al., 2017).

Ether lipids are highly abundant molecules that account for around 20% of the total phospholipid content in mammalian cells (Nagan and Zoeller, 2001; Paul et al., 2019). They make up a significant component of subcellular membranes, including the membranes of the nucleus, endoplasmic reticulum (ER), post-Golgi network and mitochondria (Nagan and Zoeller, 2001; Honsho et al., 2008). Importantly, these lipids contain varying structural and physico-chemical properties, including different head groups and fatty acyl chains. These features give rise to disparities between their distribution and function amongst tissues. High levels of ether lipids have been detected in the brain, heart, kidney, skeletal muscle and certain immune cells, including neutrophils and macrophages, whilst low levels have been reported in the liver (Lohner, 1996; Lee, 1998; Farooqui and Horrocks, 2001; Braverman and Moser, 2012). As the liver is considered a primary site for ether lipid synthesis, it has been suggested that its’ low ether lipid content is due to subsequent transport of ether lipids to other tissues via lipoproteins (Vance, 1990).

Plasmalogens are a subset of ether glycerophospholipids that bear a *cis* double bond adjacent to the ether linkage, forming a “vinyl-ether linkage” (Nagan and Zoeller, 2001; Braverman and Moser, 2012). In mammalian cells, plasmalogens are considered the most abundant and biologically active class of ether lipids, primarily consisting of palmitic (16:0), stearic (18:0) or oleic (18:1) alkenyl chains at the *sn*-1 position, and polyunsaturated fatty acids (PUFA), such as linoleic acid (18:2), arachidonic acid (20:4; AA) or docosahexaenoic acid (22:6; DHA) are typically at the *sn*-2 position (Gross, 1984; Wallner and Schmitz, 2011; Braverman and Moser, 2012).

Plasmalogens were first identified in 1924, however, it was only recently that they received attention, as studies demonstrated their capabilities as potent anti-oxidants (Feulgen and Voit, 1924; Zoeller et al., 1988, 1999). The enhanced electron density and position of the vinyl-ether linkage makes it a primary target for a variety of oxidants (Engelmann, 2004). Subsequent cleavage of the vinyl-ether linkage provides additional downstream benefits, as it prevents the oxidation of PUFAs and protects unsaturated membrane lipids. This is because plasmalogen oxidation products are unable to initiate further lipid peroxidation (Khaselev and Murphy, 1999; Murphy, 2001; Engelmann, 2004). Due to their high PUFA content at the *sn*-2 position, plasmalogens are also considered key storage depots of PUFAs. These PUFAs can be cleaved and metabolised into potent second messenger molecules, such as protectins and resolvins, to induce anti-inflammatory and anti-apoptotic effects (Ford and Gross, 1989; Schwab et al., 2007; Gaposchkin et al., 2008). Later work has since described additional roles for plasmalogens, including, but not limited to, their involvement in membrane fluidity and dynamics, intracellular signalling, immunomodulation and cholesterol metabolism (Han and Gross, 1990; Mandel et al., 1998; Farooqui and Horrocks, 2001; Gorgas et al., 2006; Lessig and Fuchs, 2009; Wallner et al., 2014; Honsho et al., 2015; Rubio et al., 2018).

### Ether Lipid Synthesis

**FIGURE 2:**
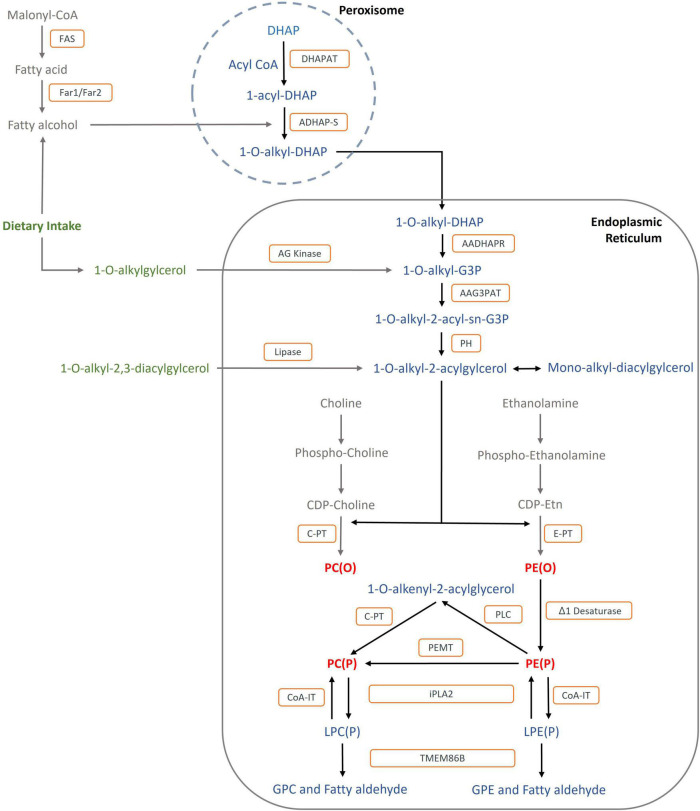
Biosynthetic and catabolic pathways of ether lipids: The formation of fatty alcohol by FAR1 and FAR2 in the peroxisome is the rate-limiting step: Metabolites are shown in blue and red: DHAP, dihydroxyacetonephosphate; G3P; glycerol-3- phosphate GPC, glycerophospho-choline; GPE, glycerophospho-ethanolamine; PC(O), Alkyl-phosphatidylcholine; PE(O), Alkyl-phosphatidylethanolamine; PC(P), PC plasmalogen, PE(P), PE Plasmalogen; LPC(P), Lyso-PC Plasmalogen; LPE(P), Lyso_PE Plasmalogen. Enzymes are shown in orange squares: DHAPAT, DHAP acyltransferase; ADHAP-S, alkyl- dihydroxyacetone phosphate synthase; AADHAPR, acyl/alkyl dihydroxyacetone phosphate reductase; AAG3PAT, acyl/alkyl-glycero-3- phosphate acyltransferase; PH, phosphohydrolase; AG kinase, alkylglycerol kinase; δδ1 Desaturase, plasmanyl-ethanolamine delta1-desaturase; C-PT, choline phosphotransferase; E-PT, ethanolamine phosphotransferase; PEMT, phosphatidylethanolamine N-methyltransferase; PLC, phospholipase C; CoA-IT, coenzyme A-independent transacylase; i-phospholipase A2, calcium independent phospholipase A2; TMEM86B, lysoplasmalogenase; FAR1, fatty acyl-CoA reductase 1; FAR2, fatty acyl-CoA reductase 2.

Ether lipid synthesis is a well characterised process that involves multiple enzymes within the peroxisome and ER (Figure 2). The pathway begins in the peroxisome with the esterification of dihydroxyacetone phosphate (DHAP) (Nagan and Zoeller, 2001). After the replacement of the acyl-chain for an alkyl-chain at the *sn*-1 position, 1-alkyl DHAP crosses to the cytosolic side of the ER where it enters the biosynthetic pathway of diacyl-phospholipids (Lee, 1998). Plasmalogens are the major end product of the biosynthetic pathway, however, platelet-activating factor (1-alkyl-2-acetyl glycerophosphoryl-choline, PAF), and the lipid moiety of distinct glycosyl-phosphatidylinositol anchored proteins are also synthesised. Paul *et al.* have reviewed the ether lipid biosynthetic pathway in more detail (Paul et al., 2019).

### Ether Lipids in Obesity

Ether lipids have been implicated in neurodegenerative disorders, cardiovascular disease (CVD), metabolic disease and some genetic disorders (Gould and Valle, 2000; Goodenowe et al., 2007; Pietiläinen et al., 2007; Graessler et al., 2009; Meikle et al., 2011). This review will focus on obesity, as it is now considered a major health burden and contributes to a range of pathologies, including CVD, insulin resistance, type 2 diabetes (T2D) and non-alcoholic fatty liver disease (NAFLD). Indeed, lipidomic studies of large human cohorts have identified decreased levels of circulating ether lipids to be associated with numerous features of metabolic diseases (Pietiläinen et al., 2007; Graessler et al., 2009; Meikle et al., 2011, 2013; Weir et al., 2013; Beyene et al., 2020).

An early lipidomic study, analysing plasma samples from monozygotic twins discordant for obesity, demonstrated that obesity was associated with increased levels of lyso-phospholipid species, which possess some pro-inflammatory effects, and a concurrent decrease in ether lipids, independent of genetic factors (Pietiläinen et al., 2007). Another study, analysing the plasma lipidome of over 1,000 individuals from Mexican-American families, observed similar results. Several ether lipid species were negatively associated with body mass index, independent of age, sex, systolic blood pressure, 2 h post-load glucose plasma levels and smoking status (Kulkarni et al., 2013). Reduced ether lipids have also been implicated in hypertension, NAFLD, pre-diabetes, T2D and ageing (Puri et al., 2009; Meikle et al., 2013; Weir et al., 2013; Paul et al., 2019).

Peroxisomes are membrane bound organelles that perform multiple functions, including ether lipid synthesis, reactive oxygen species (ROS) metabolism, fatty acid oxidation and cholesterol transport (Cipolla and Irfan, 2017). Deficiencies in the peroxisomal membrane protein Pex11a, involved in peroxisome maintenance and proliferation, reduced plasmalogen levels and caused dyslipidemia and obesity in mice (Chen et al., 2018). Additional preclinical studies have made similar observations, linking peroxisomal dysfunction, characterised by reduced levels of plasmalogens, with various metabolic pathologies including dyslipidemia, obesity, NAFLD and T2D (Cipolla and Irfan, 2017; Park et al., 2019b). Whilst the mechanisms underlying these associations remain unclear, it has been postulated that a reduction in ether lipids contributes to the disease pathology through multiple pathways, including the disruption of cellular membranes, increased oxidative stress, ER stress and inflammation. These mechanisms have been reviewed previously, and whilst they offer some insights, it is becoming increasingly apparent that the physiological roles of ether lipids are likely to be specific to a given tissue. Accordingly, investigators have begun to explore the composition of lipids in adipose tissues and subsequently uncovered novel roles for plasmalogens in the regulation of adiposity.

## *In vitro* and *in vivo* Studies

### Lipidomic Profiling of Adipose Tissue

Adipose tissue plays a central role in regulating energy metabolism and homeostasis. There are three distinct types of adipose tissue; white (WAT), brown (BAT) and beige adipose tissue. WAT is comprised of uniocular lipid droplets and is involved in storing excess energy in the form of neutral lipids, such as triacylglycerols (TG), that can be remobilised in times of energy deficiency (Leiria and Tseng, 2020). Conversely, BAT has multilocular lipid droplets and is highly metabolically active, driving non-shivering thermogenesis through the oxidation of fatty acids to generate heat (Park et al., 2019a). Beige adipocytes occur as clusters within WAT depots and are inducible, often in response to prolonged cold exposure (Leiria and Tseng, 2020). As a result, beige adipocytes are able to develop a BAT-like phenotype, giving rise to their mixed BAT and WAT functions as well as multilocular/unilocular morphology. Importantly, brown and beige adipocytes are enriched in mitochondria and express uncoupling protein 1 (UCP1), a mitochondrial membrane protein that dissociates oxidative phosphorylation from ATP production (Fedorenko et al., 2012). Lipidomic profiling of the different adipose tissues and depots has provided enormous insight into their unique composition and function.

Hoene et al. (2014) comprehensively examined the lipidome of BAT and subcutaneous WAT (SAT) in male and female mice. They demonstrated a pronounced difference in the lipid profiles of the adipose tissues, as well as a distinct sex-dependent difference in BAT. More specifically, they observed that phospholipid classes, including phosphatidylethanolamine (PE) and phosphatidylcholine (PC) ether lipids, were elevated in BAT compared to SAT (Hoene et al., 2014). Conversely, TG and diacylglycerol (DG) species were higher in SAT compared to BAT (Hoene et al., 2014). These differences likely reflect the metabolic activity and function of each tissue, as phospholipids regulate UCP1 within mitochondrial inner membranes of thermogenic BAT cells, whilst TG species are typically stored in WAT (Leiria and Tseng, 2020). Despite the lower levels of ether lipids in SAT compared to BAT, lipidomic analysis of human WAT reported that plasmalogens are still highly abundant relative to other lipid species (Lange et al., 2021). Findings showed that PE plasmalogens were the most abundant species containing PUFA as well as the fourth most abundant lipid class overall. Interestingly, PE plasmalogen levels were around 3 times higher than PE-diacyl species (Lange et al., 2021). Interestingly, another study that conducted lipidomic analysis on primary brown, white and beige adipocytes *in vitro* revealed contrary results (Schweizer et al., 2019). White adipocytes exhibited higher amounts of PE and PC ether lipids as well as lipid species containing long-chain PUFA compared to the beige and brown adipocytes. The authors suggested that this increase may reflect a protective adaptation of white adipocytes to mitigate the elevated production of ROS in the obese mice. In addition, the same group reported that brown adipocytes had a significant increase in cardiolipins (CL) compared to white and beige adipocytes (Schweizer et al., 2019). As CL are major constituents of mitochondrial membranes, these findings likely reflect the increased abundance of mitochondria within brown adipocytes compared to white adipocytes. The lipidomic profiles of brown and beige adipocytes were considered comparable (Schweizer et al., 2019).

Whilst our understanding of the adipose lipidome remains limited, particularly in relation to ether lipids, research has begun to explore the apparent remodelling of adipose tissue in the setting of obesity. Multiple studies have now comprehensively analysed the lipidomic signatures of adipose tissue from obese and lean individuals (Pietiläinen et al., 2011; Lange et al., 2021). One study conducted by Pietiläinen et al. (2011) revealed unique changes in the composition of ether lipids in twin pairs discordant for obesity (Pietiläinen et al., 2011). Their results demonstrated that the adipose tissue of the obese twins presented with increased levels of PUFA-containing ether lipids, and a proportional decrease in phospholipids containing shorter and more saturated fatty acids, compared to that of the lean twins (Pietiläinen et al., 2011). As the ether lipid and diacyl-phospholipid biosynthetic pathways are linked, these findings suggest that there is a preferential flux through the ether lipid pathway, and a concurrent decrease through the diacyl-phospholipid pathway. This shift between pathways may be linked to the important role of plasmalogens in facilitating membrane remodelling of enlarged adipocytes during obesity (Pietiläinen et al., 2011; Lange et al., 2021).

Lipidomic analyses of SAT and visceral adipose tissue (VAT) from lean and obese individuals offered greater insights into the composition of adipose tissue ether lipids in obese individuals (Lange et al., 2021). Specifically, the study revealed that higher amounts of PC plasmalogens with long-chain PUFA were characteristic of obese SAT depots, whilst PE plasmalogen species accumulated in obese VAT depots. Similar findings were also observed in women with insulin resistance, which is a hallmark of obesity (Wentworth et al., 2016). Wentworth et al. (2016) reported that PC ether lipids were more abundant in the SAT depots of women with insulin resistance compared to the VAT depots. Together, these findings demonstrate that the differences in lipid composition appear to be highly depot specific. SAT is considered a more metabolically healthy adipose tissue, in part due to its ability to undergo browning, and therefore has the potential to develop a BAT-like phenotype (Chechi et al., 2018). Conversely, VAT is known to be a major risk factor for cardiometabolic disease and has been linked to hyperglycaemia, hyperinsulinemia, hypertriglyceridaemia and impaired glucose tolerance (Ibrahim, 2010). Interestingly, PE and PC phospholipids have specific and opposing effects on membrane stability, as PE lipids promote membrane rigidity whilst PC lipids maintain membrane fluidity (Harayama and Riezman, 2018). It has been suggested that the expansion of adipose tissue associated with obesity triggers remodelling of membrane phospholipids in an effort to maintain membrane composition and function (Lange et al., 2021). However, the specific role of PE and PC plasmalogen species in this context remains elusive. Furthermore, it is unclear whether these findings reflect increased endogenous synthesis of ether lipids in WAT or uptake of ether lipids from the circulation.

Following on from their early work, Pietiläinen et al. (2011) performed lipidomic analysis on the SAT of healthy and morbidly obese weight-discordant twins (Pietiläinen et al., 2011). Surprisingly, they demonstrated that unlike in the obese twins, remodelling of PUFA-plasmalogens was considerably reduced in the morbidly obese twins. These findings suggest that the protective adaptations induced by the onset of obesity are lost as adiposity increases. One plausible explanation involves the enzyme calcium independent phospholipase A2 (iPLA2), which hydrolyses plasmalogens into lyso-plasmalogens (Paul et al., 2019). Importantly, iPLA2 is known to be elevated in the setting of obesity and has been shown to contribute to diet-induced weight gain, adipocyte hypertrophy and insulin resistance, via changes in fatty acid oxidation and mitochondrial content *in vivo* (Garces et al., 2010; Mancuso et al., 2010). Based on the current literature, it is likely that iPLA2 levels increase during obesity which contributes to the reduction in plasmalogen levels. This would result in a subsequent decrease in mitochondrial content and function, as well as exacerbate diet-induced obesity. Furthermore, deacylation of plasmalogens results in the release of PUFAs at the *sn*-2 position. PUFAs are highly susceptible to lipid peroxidation, resulting in the formation of toxic lipid peroxyl radicals and hydroperoxides (Antonio et al., 2014). One example of this is oxidation of AA. AA can be oxidised into precursors of multiple eicosanoids, including thromboxane, prostaglandins, and leukotrienes, which have potent inflammatory functions (Farooqui et al., 1995; Marzo, 1995). Elevated levels of these lipid mediators could contribute to the systemic inflammation associated with morbid obesity. Indeed, this rapid break down of plasmalogen species via iPLA2 would likely exceed the rate of endogenous plasmalogen synthesis, resulting in the overall decrease of PUFA-containing plasmalogens that was observed. It is important to note that it remains unclear whether iPLA2 is plasmalogen specific, or it hydrolyses other structurally similar lipids concurrently. Furthermore, due to the complex nature of lipid metabolism, the regulatory mechanisms responsible for this obesity-induced lipid dysregulation are likely to involve multiple intrinsic and overlapping pathways.

### Functional Roles of Ether Lipids in Adipose Tissue

Studies by Brites et al. (2011) and Park et al. (2019b), have been critical in developing our understanding of the functional roles of ether lipids, particularly plasmalogens, within adipocytes. Using *in vivo* and *in vitro* models of impaired peroxisomal function to induce plasmalogen deficiency, Brites et al. (2011) revealed a crucial role for plasmalogens in facilitating lipid droplet formation. Mice deficient in plasmalogens, via knockout of the peroxisomal factor 7 (Pex7), presented with extremely reduced epididymal, inguinal, retroperitoneal and subscapular WAT depots, whilst the brown adipocytes had abnormally small lipid droplets (Brites et al., 2011). Interestingly, dietary supplementation with the plasmalogen precursor alkylglycerols, rescued plasmalogen levels and normalised the size and number of lipid droplets in both the BAT and WAT of the plasmalogen deficient mice (Brites et al., 2011). These results suggest a role for plasmalogens in lipid droplet formation and maintenance. Consistent with this, additional studies demonstrated that plasmalogen-deficient mouse embryonic fibroblasts (MEFs) had fewer and smaller lipid droplets when compared to control MEFs (Brites et al., 2011). Similarly, treatment with alkylglycerols restored the number and volume of lipid droplets, further supporting a role for plasmalogens in the regulation of lipid droplet homeostasis.

Studies have demonstrated that lipid droplets also mediate ER stress (Hapala et al., 2012). ER stress is a common feature of obesity that results in the disruption of protein folding and synthesis (Basseri and Richard, 2011). Researchers have shown that an increase in lipid droplet biogenesis, often during obesity, induces ER stress. The primary mechanisms thought to drive this association was a combination of reduced phospholipid synthesis and an up-regulation of TG synthesis (Basseri and Richard, 2011). As plasmalogens have been implicated in lipid droplet formation, it is likely that the known reduction of plasmalogens caused by obesity in directly contributes to the progression of ER stress. This is supported by recent work by Ogawa and colleagues who discuss a potential link between elevated ER stress, mitochondrial dysfunction and inflammation with reduced levels of PE plasmalogens in patients with bipolar disorder (Ogawa et al., 2020). Additionally, an *in vivo* study utilising peroxisome-deficient *Pex2* knockout mice, demonstrated that functional peroxisomes are critical for the prevention of chronic ER stress (Kovacs et al., 2009). Whilst the study did not link peroxisomal function with plasmalogen levels directly, combining these results with their known role as potent anti-oxidants suggests a protective role of plasmalogens against oxidative stressors. Further exploration into the specific role of ether lipids in the setting of ER stress is required.

Recently, peroxisomal lipid metabolism, and subsequent ether lipid synthesis, has been shown to facilitate thermogenesis via the regulation of mitochondrial dynamics. Inhibition of peroxisome biogenesis via the WAT-specific deletion of the peroxisomal biogenesis factor Pex16 *in vivo*, decreased mitochondrial DNA content and impaired mitochondrial function in brown and beige adipocytes (Park et al., 2019b). As a result, the knockout mice presented with severe cold intolerance and reduced thermogenesis. Furthermore, when placed on a high fat diet, the knockout mice had significantly increased fat mass and body weight compared to control mice on the same diet, demonstrating diet-induced obesity. Subsequent dietary supplementation of alkylglycerols was able to restore plasmalogen levels, mitochondrial morphology and cold sensitivity in these mice (Park et al., 2019b). Together, these findings suggest that peroxisomal synthesis of ether lipids is important for regulating mitochondrial dynamics and thermogenesis. Using an alternative model of plasmalogen deficiency, via knockdown of the endogenous ether lipid synthesis enzyme glyceronephosphate O-acyltransferase (GNPAT), researchers observed similar impairments in mitochondrial fission and oxygen consumption in BAT stromal vascular fraction (SVF) *in vitro* (Park et al., 2019b). These findings are particularly intriguing as attenuation of *Gnpat* inhibits ether lipid synthesis but does not impede peroxisomal function. This suggests that the observed mitochondrial dysfunction may occur in response to reduced ether lipids, rather than peroxisomal dysfunction *per se*.

The underlying mechanisms by which ether lipids regulate thermogenesis are starting to become more clear, as a study has now demonstrated that mitochondrial membrane lipids, including plasmalogens, mediate thermogenesis through crosstalk between organelles, including the nucleus and peroxisomes (Jiménez-Rojo and Riezman, 2019). Park et al. (2019b) demonstrated that thermogenic stimuli increased peroxisome proliferation via activation of PRDM16 (PR domain containing 16). This subsequently increased plasmalogen content within the mitochondria, which promoted mitochondrial fission and potentiated free fatty acid (FFA)-induced uncoupling and energy expenditure in BAT (Park et al., 2019a). The known role of the vinyl-ether linkage to foster non-lamellar lipid structures and regulate membrane dynamics supports this role for plasmalogens. However, the extent to which plasmalogens mediate mitochondrial fission and morphology remains unclear. It has been speculated that it may involve effects on mitochondrial localisation and/or activity of fission factors (Park et al., 2019a).

PUFAs have been shown to promote thermogenesis via cell signalling (Sadurskis et al., 1995; Fan et al., 2019). As plasmalogens are rich in PUFAs, this may be an additional mechanism by which they promote thermogenesis in BAT. An *in vivo* study exploring the effect of PUFAs on non-shivering thermogenesis demonstrated that mice on a high PUFA diet exhibited an improved thermogenic capacity of BAT compared to mice fed a diet with standard fat content (Sadurskis et al., 1995). Interestingly, these effects appear to be specific to omega-3 PUFA, such as eicosapentaenoic acid (EPA) and DHA. EPA has been shown to promote BAT differentiation, increase UCP1 gene expression and decrease adiposity in mice (Ghandour et al., 2018). Surprisingly, omega-6 PUFA, such as AA, inhibit the conversion of white to beige adipocytes and favour obesity (Pisani et al., 2017). As plasmalogens carry both omega-3 and omega-6 PUFA at the *sn*-2 position, understanding their composition in BAT and WAT may offer further insights into their functional roles in these tissues.

### Ether Lipids in Infant Adipose Tissue

A recent study has identified a unique role of ether lipids in breast milk in the prevention of obesity in early life (Yu et al., 2019). Breast milk is a rich source of lipids and is essential for adipose tissue physiology (Rosen and Spiegelman, 2014; Koletzko, 2016). TG(O)’s are the primary ether lipid in breast milk and have been shown to not only facilitate the development of beige adipocytes in neonatal mice, but also impede white adipocyte accumulation (Yu et al., 2019). In this study, investigators increased the alkylglycerol intake of neonatal mice by 20% and observed an increase in mitochondrial content, UCP1 transcription and beiging area of the SAT of the treated mice. Conversely, adipocyte size and TG content was markedly reduced in the treated mice when compared to the control mice that did not receive alkylglycerol supplementation. This effect of alkylglycerols on infant adipocytes was proposed to occur via adipose tissue macrophage signalling. Adipose tissue macrophages (ATMs) are important for lipid and energy metabolism as well as mitochondrial function in adipocytes (Li et al., 2020). This study demonstrated that the alkylglycerols were metabolised by ATMs, triggering an increase in PAF levels and the subsequent release of IL-6 (Yu et al., 2019). IL-6 is an interleukin that activates the transcription of adipocyte STAT3, which facilitates beige adipocyte development (Yu et al., 2019). Interestingly, a lack of alkylglycerol intake during infancy led to a premature loss of beige adipocytes and an increase in fat accumulation (Yu et al., 2019). Whilst further exploration is required, this novel study highlights the importance of breast milk alkylglycerols in promoting healthy adipose tissue development in early life.

Later in life, ATMs perform contrasting roles, as they maintain metabolic homeostasis, but also contribute to the aetiology of obesity through non-resolving inflammation (Morgan et al., 2021). Progressive lipid accumulation within macrophages drives a switch between the polarization of anti-inflammatory M2 ATMs to pro-inflammatory M1 ATMs (Prieur et al., 2011). In the setting of obesity, M2 ATMs are critical for the removal of necrotic-like adipocytes, as well as facilitating lipid storage in WAT (Cinti et al., 2005; Cox et al., 2021). A recent study revealed that treatment of M1 and M2 bone marrow-derived macrophages (BMDM) with exogenous fatty acids caused an increase in TG and cholesterol ester (CE) species in M1 macrophages, whilst a greater increase in PE and PC ether lipids was observed in M2 macrophages (Morgan et al., 2021). Mechanistically, M2 macrophages express higher levels of *Gnpa*t and *Far1*, the enzymes involved in the first and rate limiting steps of endogenous ether lipid synthesis, respectively (Jha et al., 2015). Together, these findings suggest that ether lipids may play an important role in promoting the anti-inflammatory phenotype of M2 macrophages. Furthermore, the anti-oxidant properties of the vinyl-ether linkage may be a key mechanism by which plasmalogens reduce inflammation and oxidative stress, thereby negating some of the effects of obesity. Based on these findings it is evident that promoting M2-like ATMs may be important for reducing the influx of obesity-associated inflammatory cytokines and mediators, driven by M1-like ATMs (Zeyda et al., 2007).

## Therapeutic Potential of Ether Lipids for Attenuating Obesity

As the protective effects of ether lipids have become more clear, considerable work has begun to explore the potential therapeutic effects of modulating ether lipid levels to attenuate obesity and its subsequent complications. There are two major approaches for modulating endogenous ether lipid levels: 1) genetic modulation of enzymes involved in the ether lipid metabolism and 2) supplementation with their metabolic precursors, such as alkylglycerols (1-O-alkylglycerol or 1-O-alkyl-2,3- diacylglycerol) (Figure 2).

Utilising a *Gnpat* knockout mouse model to induce plasmalogen deficiency, Jang et al. (2017) demonstrated that upon feeding of a high fat diet, mice were more susceptible to hepatic lipid accumulation, adipose tissue inflammation and high fat diet-induced insulin resistance compared to wild type mice (Jang et al., 2017). In contrast, alkylglycerol supplementation has been shown to increase plasmalogen levels in cells, animals and humans, resulting in the suppression of some features of metabolic diseases (Das et al., 1992; Brites et al., 2011). A pivotal study using alkylglycerol supplementation in a mouse model of diet-induced obesity and insulin resistance demonstrated that 8 weeks of alkylglycerol treatment decreased body weight, serum TG, cholesterol and fasting insulin levels (Zhang et al., 2013). It is possible that the reduction in body weight may be linked to increased lipolysis of WAT in an attempt to release more FFA to facilitate thermogenesis. An *in vitro* study has also shown that alkylglycerols are effective at reducing oxidative stress, a hallmark of obesity and subsequent metabolic diseases (Zoeller et al., 2002). Cultured human pulmonary arterial endothelial cells (PAEC) were supplemented with alkylglycerols for 6 days and exhibited a two-fold increase in plasmalogen levels. Importantly, the PAEC were protected against hypoxia and other stressors linked to reactive oxygen species (Zoeller et al., 2002).

Shark liver oil is a natural product rich in alkylglycerols. A recent preliminary clinical study assessed the ability of shark liver oil supplementation to modulate plasma and immune cell plasmalogen levels in overweight or obese men (Paul et al., 2021b). The study reported significant changes in the levels of multiple ether lipid species in plasma and circulating white blood cells, including 59% and 15% increases in PE plasmalogens in the plasma and white blood cells, respectively (relative to total PC levels). Furthermore, total cholesterol, TG levels and the inflammatory marker C-reactive protein all decreased (Paul et al., 2021b). These results support the concept that shark liver oil enriches plasma and cellular plasmalogens to provide protection against obesity-related dyslipidaemia and inflammation.

**FIGURE 3:**
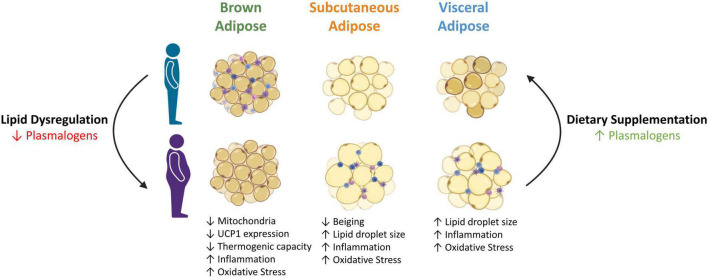
Possible role of plasmalogens in adipose tissue metabolism and function in the setting of obesity. Obesity is characterised by enlarged adipocytes and severe lipid dysregulation, including reduced plasmalogens. Decreased plasmalogen levels impedes the normal function and size of adipocytes, further exacerbating the metabolic complications associated with obesity. Conversely, increasing plasmalogen levels via dietary supplementation appears to revert the effects of obesity on adipocytes by restoring their size and function. UCP1, uncoupling protein 1. Created with Biorender.com (2021).

A recent study examined the impact of supplementation of an alkylglycerol mix on the plasma and various tissues, including the liver, VAT and skeletal muscle of mice *in vivo* (Paul et al., 2021a). After 1 to 12 weeks of the mixed alkylglycerol treatment, PE and PC ether lipids, including plasmalogens, progressively increased in the VAT. These results demonstrate the ability of dietary alkylglycerols to penetrate the adipose tissue and successfully incorporate into the ether lipid biosynthetic pathway (Paul et al., 2021a). As discussed previously, dietary supplementation with alkylglycerols rescued adipocyte morphology and reduced diet-induced obesity in mice with plasmalogen deficiencies (Figure 3) (Brites et al., 2011). Whilst obesity has been the focal point for this review, it is important to note that alkylglycerols have also proven effective in the treatment of NAFLD, genetic peroxisomal disorders and CVD (Das et al., 1992; Wood et al., 2011; Rasmiena et al., 2015; Parri et al., 2016; Jang et al., 2017). Furthermore, natural plasmalogens such as scallop-purified and chick-skin PE plasmalogens have been successful at increasing plasmalogen levels in human and animal studies (Maki et al., 2009; Tandy et al., 2009; Mawatari et al., 2012, 2020).

In summary, ether lipids are important biological molecules with functional roles within adipose tissue. Numerous studies now demonstrate a clear relationship between circulating ether lipids and obesity, as reduced plasmalogen levels are apparent in obese individuals and facilitate diet-induced obesity *in vivo.* Combining these studies further suggests that the increased oxidative stress and inflammation associated with obesity promotes the dysregulation of ether lipids in adipose tissue. As lipid metabolism is highly complex, it remains unclear whether the reduced ether lipids drive obesity, or obesity drives the observed decrease in ether lipids. Importantly, this work highlights potential functional roles for ether lipids in the protection against diet-induced obesity. Thus, increasing plasmalogen levels may be beneficial in the attenuation of obesity and its complications via the promotion of thermogenesis, antioxidant effects and cellular signalling to reduce inflammation.

## Author Contributions

YLS: writing—original draft preparation. YLS, SP, ACC, and PJM: writing—manuscript revision. SP, ACC, and PJM: supervision. All authors have read and agreed to the published version of the manuscript.

## Conflict of Interest

PJM is inventor on a patent; WO 2021/007623 A1; Title: Compositions for maintaining or modulating mixtures of ether lipid molecules in a tissue of a human subject. The remaining authors declare that the review was conducted in the absence of any commercial or financial relationships that could be construed as a potential conflict of interest.

## Publisher’s Note

All claims expressed in this article are solely those of the authors and do not necessarily represent those of their affiliated organizations, or those of the publisher, the editors and the reviewers. Any product that may be evaluated in this article, or claim that may be made by its manufacturer, is not guaranteed or endorsed by the publisher.
